# A Rare Case of Quincke's Disease With an Unknown Trigger

**DOI:** 10.1002/ccr3.70270

**Published:** 2025-02-24

**Authors:** Katrina J. Villegas, Mazhar Mustafa, Shivanck Upadhyay, John Kashani

**Affiliations:** ^1^ Department of Internal Medicine St. Joseph's University Medical Center Paterson New Jersey USA; ^2^ Department of Pulmonary and Critical Care Medicine St. Joseph's University Medical Center Paterson New Jersey USA; ^3^ Department of Emergency Medicine St. Joseph's University Medical Center Paterson New Jersey USA

**Keywords:** Quincke's disease, Type I hypersensitivity, unknown trigger, uvular angioedema

## Abstract

Quincke's disease is a rare presentation of angioneurotic edema of the uvula alone, likely a Type I immediate hypersensitivity reaction. We raise awareness of this rare but life‐threatening case presenting as throat tightness, without rash or facial angioedema, treated with corticosteroids, antihistamines, and β2‐agonist nebulization.

## 1 Case Presentation

**FIGURE 1:**
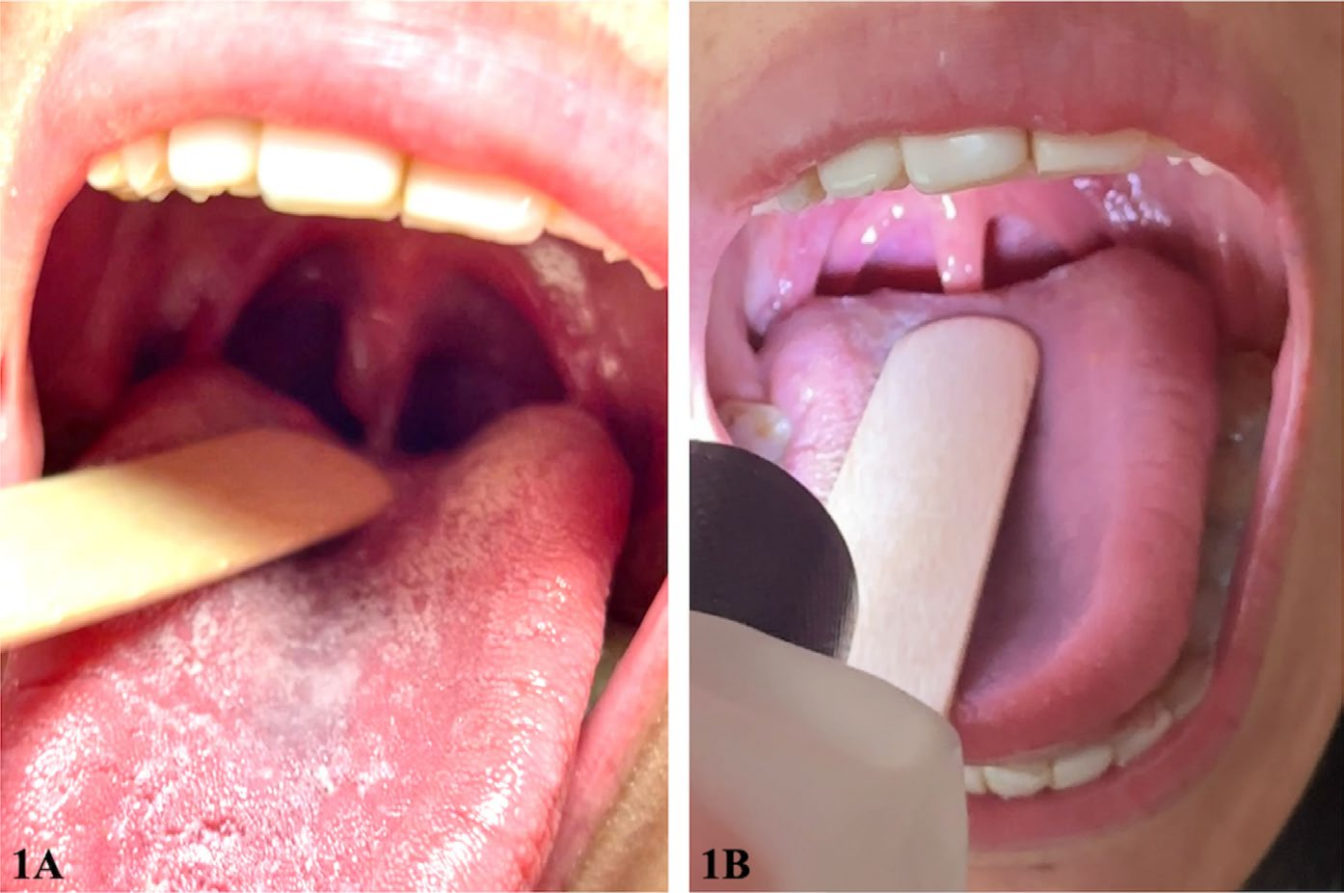
(A) (*Left*) Clinical image showing Quincke's disease, globular swelling of the uvula. (B) (Right). Clinical image showing resolution of the angioedema of the uvula within 24 h.

This is a 33‐year‐old female with a medical history of childhood asthma and seasonal allergy who presented to the emergency department with complaints of tightness of the throat that has been worsening over the past 2 days, associated with chest tightness and shortness of breath, difficulty swallowing, and a muffled low‐pitch voice. She denied any pain, fever, trauma, or any known food or drug allergy. She has not identified any specific allergen that possibly brought about her symptoms. She also denied having similar symptoms in the past. Upon physical examination, she was noted to have an edematous and globular uvula (Figure 1A, Left). There was no skin rash or wheals, no trismus, no base of tongue edema, no stridor, and no wheezing. She received dexamethasone 10 mg IV, diphenhydramine 25 mg IV, and albuterol nebulization, after which she reported improvement of her symptoms. A computed tomography scan of the neck with contrast showed no evidence of a mass in the soft tissues of the neck. She was admitted to the medical ICU for close monitoring of her airway. She was discharged with a note of improvement of the isolated uvular edema (Figure 1B, Right) and improvement of her symptoms. She was prescribed tapered doses of steroids and advised to avoid possible food and drug allergens.

Given the patient's stable condition upon discharge, further studies for pneumoallergens and trophoallergens were not pursued at that time. The patient was advised to follow‐up as an outpatient for these allergy tests; however, she currently lacks insurance coverage and is in the process of obtaining charity care, which has delayed her follow‐up with a primary care physician or an allergologist.

## 2 Discussion

Quincke's disease is a rare presentation of an angioneurotic edema, an upper airway edema isolated to the uvula. Patients usually complain of a globus sensation of the throat, hoarseness, coughing, gagging, drooling, or difficulty breathing, but without fever or rash [1]. This may be due to an anaphylactic reaction to food allergens; hereditary angioedema, inhalational exposure, drug sensitivity, and trauma to the area have also been implicated. Although our patient could not identify a specific trigger, the most common underlying etiology is a Type I immediate hypersensitivity reaction found in atopic states [2, 3], such as in our patient who has a history of childhood asthma and seasonal allergy. In recurrent episodes, C1 esterase inhibitor deficiency or hereditary angioedema should be suspected [3]. As this has the potential to be life‐threatening, management is primarily focused on ensuring a patent airway. Medical treatment includes intravenous antihistamines, intravenous or inhalational corticosteroids, and β2‐agonist nebulization. Among the steroids, dexamethasone is the agent of choice because of its potent anti‐inflammatory properties and long half‐life [1, 2]. Epinephrine is considered in severe cases. Patients should be educated to avoid potential allergens, to promptly identify similar symptoms, and to proceed to the ED if symptoms occur.

## Author Contributions

**Katrina J. Villegas:** data curation, formal analysis, visualization, writing – original draft, writing – review and editing. **Mazhar Mustafa:** conceptualization, data curation, formal analysis, supervision. **Shivanck Upadhyay:** conceptualization, data curation, formal analysis, supervision. **John Kashani:** data curation, formal analysis, resources.

## Consent

As this is a clinical case image report, written consent was obtained for the purpose of this paper.

## Conflicts of Interest

The authors declare no conflicts of interest.

## Data Availability

Data openly available in a public repository that issues datasets with DOIs.

## References

[1] G. A. Sanchez , M. Boot , and A. Lathif , “Quincke's Disease: An Unusual Pathology,” Journal of Surgical Case Reports 2023, no. 3 (2023): rjad085, 10.1093/jscr/rjad085. 36896152 PMC9989133

[2] A. Chandran , P. Sakthivel , and A. S. Chirom , “Quincke's Disease,” BMJ Case Reports 12, no. 9 (2019): e231967, 10.1136/bcr-2019-231967. PMC6738731 31511276

[3] R. R. Kamath and S. J. Rai , “Quincke's Disease: A Case Report,” Egyptian Journal of Otolaryngology 36, no. 1 (2020): 44, 10.1186/s43163-020-00048-8.

